# Necroptotic debris including damaged mitochondria elicits sepsis-like syndrome during late-phase tularemia

**DOI:** 10.1038/cddiscovery.2017.56

**Published:** 2017-09-25

**Authors:** Anju Singh, Sivakumar Periasamy, Meenakshi Malik, Chandra Shekhar Bakshi, Laurie Stephen, Jeffrey G Ault, Carmen A Mannella, Timothy J Sellati

**Affiliations:** 1 Center for Immunology and Microbial Disease, Albany Medical College, Albany, NY 12208, USA; 2 Ampersand Biosciences LLC, Saranac Lake, NY, 12983, USA; 3 Wadsworth Center, New York State Department of Health, Albany, NY 12201, USA

## Abstract

Infection with *Francisella tularensis* ssp. *tularensis* (*Ft*) strain SchuS4 causes an often lethal disease known as tularemia in rodents, non-human primates, and humans. *Ft* subverts host cell death programs to facilitate their exponential replication within macrophages and other cell types during early respiratory infection (⩽72 h). The mechanism(s) by which cell death is triggered remains incompletely defined, as does the impact of *Ft* on mitochondria, the host cell’s organellar ‘canary in a coal mine’. Herein, we reveal that *Ft* infection of host cells, particularly macrophages and polymorphonuclear leukocytes, drives necroptosis via a receptor-interacting protein kinase 1/3-mediated mechanism. During necroptosis mitochondria and other organelles become damaged. *Ft*-induced mitochondrial damage is characterized by: (i) a decrease in membrane potential and consequent mitochondrial oncosis or swelling, (ii) increased generation of superoxide radicals, and (iii) release of intact or damaged mitochondria into the lung parenchyma. Host cell recognition of and response to released mitochondria and other damage-associated molecular patterns engenders a sepsis-like syndrome typified by production of TNF, IL-1*β*, IL-6, IL-12p70, and IFN-*γ* during late-phase tularemia (⩾72 h), but are absent early during infection.

## Introduction

*Francisella tularensis* subspecies *tularensis* (*Ft*), a highly virulent facultative intracellular bacterium, is the causative agent of tularemia, an acute and sometimes fatal disease. A consequence of infection of host cells by *Ft* is the induction of host cell death (HCD).^1^ Bacterial pathogens use various strategies to manipulate HCD to ensure their survival and facilitate replication. Although the mechanisms whereby HCD pathways are manipulated depends on the nature of the pathogen and the cell type involved, the following three pathways often are targeted by bacteria, the (i) mitochondrion-dependent cell death pathway, (ii) inflammasome-dependent cell death pathway and (iii) NF-*κ*B-dependent prosurvival pathway.^2–4^ Apoptosis or programmed HCD occurs via an extrinsic or intrinsic pathway. While the extrinsic pathway is activated upon stimulation of transmembrane death receptors (for example, FasL, TNFR1, and Apo2/Apo3), leading to the activation of effector caspase-3 and -7, the intrinsic pathway is initiated through the release of signaling factors from mitochondria. Disruption of outer mitochondrial membrane integrity is mediated by proapoptotic Bax and Bak, followed by the release of cytochrome *c*, activation of caspase-9, and collapse of mitochondrial transmembrane potential.^3^

While apoptosis is generally ‘immunologically silent’, necrosis is accompanied by secretion of proinflammatory cytokines and the extracellular release of a cell’s cytosolic contents.^5^ Necrosis is characterized by disorganized DNA hydrolysis, and plasma membrane permeabilization leading to release of high-mobility group B1 (HMGB1) protein, heat-shock proteins, and mitochondria and mitochondrial DNA (mtDNA) into the extracellular milieu.^6,7^ More recently, a form of programmed necrosis, called necroptosis, has been described.^8–11^ Necroptosis is normally initiated via Fas or TNF death receptors, leading to activation of receptor-interacting protein kinase 1 or 3 (RIP1/RIP3).^12–14^

If not cleared due to impaired efferocytosis, apoptotic cells undergo secondary necrosis, a proinflammatory event associated with cell membrane disruption and the release of cytosolic and nuclear contents into the extracellular milieu.^15^ Secondary necrotic cells, particularly macrophages and polymorphonuclear leukocytes (PMNs), represent an important source of damage-associated molecular patterns (DAMPs) and a potential pathogenic mechanism driving acute and intense lung inflammation. Recently, it was demonstrated that mitochondria released from cells during RIP1-dependent necroptosis serve as *bona fide* danger signals.^12^ Engulfment of mitochondria by macrophages and dendritic cells modulates macrophage secretion of cytokines and induction of dendritic cell maturation, respectively. It has been suggested that *Ft* infection induces macrophage death in a manner resembling the intrinsic apoptotic pathway;^16^ however, this work confined its focus to murine macrophage-like J774.1 cells infected with *Ft* live vaccine strain (LVS). More recently, Doyle *et al*.^17^ used mouse bone marrow-derived monocytes (BMDMs), but focused their attention on the ability of TolC protein to delay induction of apoptosis by *Ft* LVS.^17^ *Ft* SchuS4 was shown to induce extensive apoptosis in a mouse model of respiratory tularemia, resulting in the release of DAMPs such as HMGB1 and S100A9.^18,19^ To date, whether and the mechanism whereby *Ft* infection induces apoptotic cells to undergo secondary necrosis and release organellar DAMPs (that is, mitochondria) has been unexplored.

Herein, we demonstrate that early during infection (⩽72 h), *Ft-*induced HCD in BMDMs proceeds via both the extrinsic (caspase-8-mediated) and intrinsic (caspase-9-mediated) apoptotic pathways, both of which converge on caspase-3/7. Following a delay in apoptosis, these cells rapidly undergo programmed secondary necrosis (i.e., necroptosis) with subsequent release of cell debris and DAMPs, including mitochondria. Electron microscopy revealed that mouse macrophages engulf mitochondria released from necroptotic cells in the inflamed lung. Importantly, it is direct recognition of and response to mitochondria and likely other DAMPS (e.g., HMGB1 and S100A9) and not *Ft* itself that elicits the T_H_1-oriented cytokine ‘storm’ associated with late-phase (≥72 h) respiratory tularemia.

## Results

### HCD induced early during *Ft* infection increases over the course of disease

**Figure 1:**
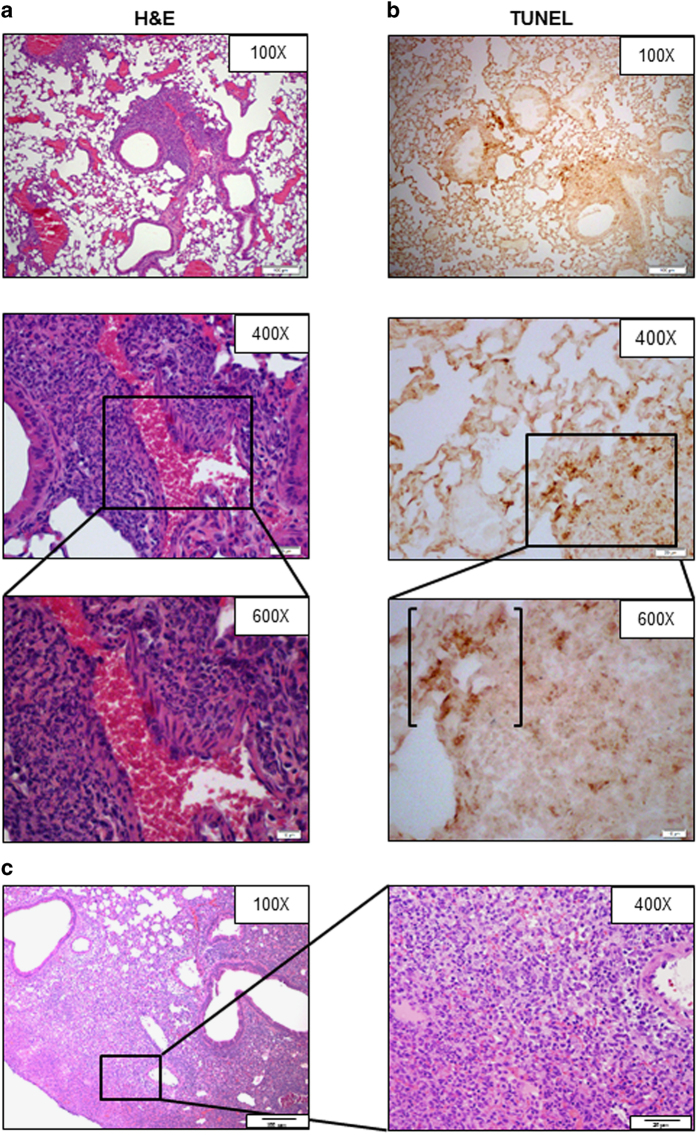
Respiratory infection with *Ft* LVS induces apoptosis and secondary necrosis. Perfused lungs from day 3 *Ft*-infected (10^3^ CFU) C57BL/6 mice were recovered and processed for histological evaluation. Paraformaldehyde-fixed, paraffin-embedded sections were stained with hematoxylin–eosin (H&E) (**a**) or TUNEL (**b**). Evidence of apoptosis and necrotic cell debris was mixed with the ground substance in the lung parenchyma. The ×600 panels represent magnifications of the boxed areas seen in the ×400 panels. Bracketed area (**b**, ×600) represents the TUNEL-positive fragmented nucleic acid released from the dead/damaged cells. Results are representative of six individual mice. (**c**) By day 6 p.i., necrotizing inflammation is a hallmark pathology of pulmonary tularemia. Note the accumulation of mixed cellular infiltrates (including PMNs, macrophages, and lymphocytes) in alveolar lumen/interstitium and necrotic areas in the lung parenchyma (×100). Inset picture: necrotic (Nec) lung parenchyma and dead/dying cells (arrow) in the vicinity of necrotic areas (×400).

Late-phase tularemia is typified by intense hypercytokinemia and severe tissue pathology. Since the host does not produce measurable levels of TNF, IL-1*β*, IL-6, IL-12p70, and IFN-*γ* in direct response to *Ft* LVS and SchuS4 (when adapted to the mammalian host)^20,21^ *in vivo* or *ex vivo* at early time points, we wondered what triggers this element of the cytokine ‘storm’ during late-phase disease. Recognizing that several subspecies of *Ft* (that is,* holarctica* and *tularensis*) and other species of the genus *Francisella* (that is, *novicida*, *Fn*) induce various forms of programmed cell death,^16,18,19,22^ we hypothesized that direct recognition of host-derived DAMPs might drive the transition from limited to fulminate T_H_1-type cytokine production. To characterize the process of cell death *in vivo*, mice either were sham inoculated or infected with *Ft* LVS and PBS-perfused lungs were harvested at day 3 and 6 post infection (p.i.) for evaluation. At day 3, peribronchiolar and perivascular ‘cuffing’ was evident and consists of an intense infiltrate of PMNs containing a few scattered macrophages (Figure 1). Histological evidence of necrotic cells and cellular debris mixed with the ground substance of the lung parenchyma was observed at higher magnification (Figure 1a, ×600, extracellular areas of eosin staining). Terminal deoxynucleotidyl transferase dUTP nick-end labeling (TUNEL) staining was strictly localized to the inflammatory lesions (Figure 1b), and while much of it was bounded by the plasma membrane of numerous infiltrating cells, there also was evidence of cell-free TUNEL staining within the extracellular milieu, evidence of fragmented nucleic acid released from dead cells (bracketed area in Figure 1b, ×600). TUNEL staining results coincide with those obtained by staining with Annexin V.^23^ Importantly, similar lung pathology at day 3 was observed following infection with *Ft* SchuS4 (data not shown).

Lung pathology was even more severe at day 6 with multiple inflammatory foci and necrotic changes, consistent with bronchopneumonia (Figure 1c). Inflammatory foci contained a mixed infiltrate of immature and mature PMNs, macrophages and lymphocytes, serous to fibrinous exudation in the alveolar space, and an accumulation of inflammatory cells in the alveolar and bronchiolar lumen. Granulomas were evident throughout the lungs as was caseous necrosis, without suppuration.

**Figure 2:**
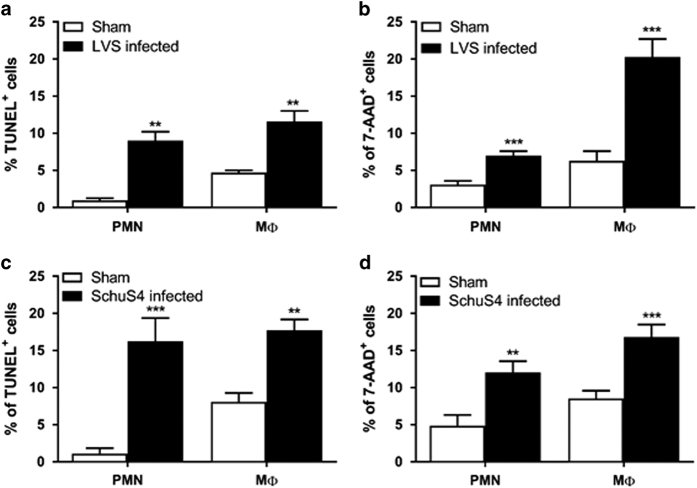
Myeloid cells undergo apoptosis/necrosis during the course of infection with *Ft*. Total lung cells were isolated from C57BL/6 mice infected with 10^3^ CFU of *Ft* LVS (**a** and **b**) or 20 CFU of SchuS4 (**c** and **d**) at day 3 p.i. The cells were stained with TUNEL (**a** and **c**) or 7-aminoactinomycin D (7-AAD) (**b** and **d**) to quantify the percentage of myeloid cells undergoing apoptosis and necrosis. The lungs of *Ft* SchuS4-infected mice were not perfused before isolation of cells as to minimize potential liquid dispersal of select agent. Data are presented as the mean±S.E.M. from three independent experiments (*n*=6 mice per group or 18 mice total). **P<0.01, and ***P<0.001.

To identify the subsets of myeloid cells undergoing apoptosis and/or necrosis at day 3, lung cells isolated from mice infected with *Ft* LVS or SchuS4 were stained with TUNEL and 7-AAD, a more specific stain for necrotic cells,^24^ and analyzed by flow cytometry. As shown in Figures 2a and b, PMNs and macrophages are the prominent cell types undergoing apoptotic and necrotic cell death in the lungs of *Ft* LVS-infected mice. The percentage undergoing cell death was significantly higher compared with cells isolated from lungs of sham-inoculated mice. These differences were reflected in the total number of cells elicited by *Ft* LVS infection as well (Table 1). The same patterns of cell death were observed in SchuS4-infected mice (Figures 2c and d and Table 2).

### Kinetic analysis reveals that the nature of HCD ‘evolves’ during late-phase disease

**Figure 3:**
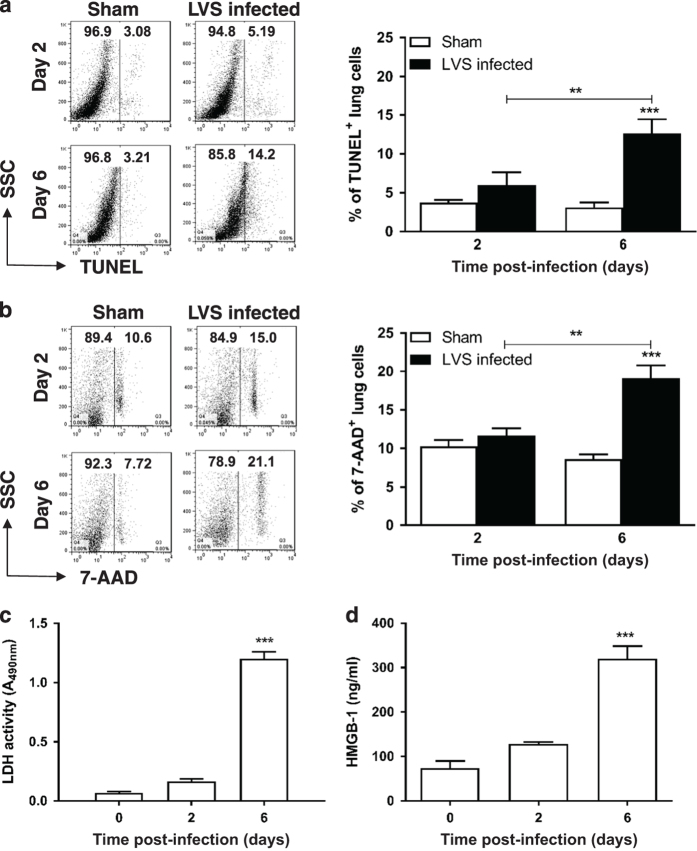
Apoptotic/necrotic cells accumulate during the course of infection with *Ft* LVS. Total lung cells were isolated from *Ft-*infected (10^3^ CFU) C57BL/6 mice at different time points and were stained with TUNEL (**a**) or 7-aminoactinomycin D (7-AAD) (**b**) to quantify the percentage of apoptotic and necrotic cells, respectively. **P<0.01 and ***P<0.001. Lactate dehydrogenase (LDH) (**c**) and HMGB1 (**d**) levels were determined in the bronchoalveolar lavage fluid (BALF) samples isolated from the *Ft*-infected mice at various time points p.i. Data are presented as the mean±S.E.M. from two independent experiments (*n*=6 mice per group or 12 mice total). ***P<0.001. All results shown were subjected to one-way analysis of variance (ANOVA) with Bonferroni’s post-test.

Histopathological analyses have demonstrated that *Ft* induces extensive necrotic changes in lung parenchyma and death of infiltrating myeloid cells.^1,25–28^ However, the nature of cell death induced by *Ft* has not been fully explored. Kinetic analysis of cell death during the early and late phase of infection revealed that while there was a slight increase in TUNEL^+^ cells at day 2, a marked increase from 6.1 to 13.3% (*P*<0.01) was observed by day 6 (Figure 3a). Similarly, Figure 3b shows an increase in the frequency of 7-AAD^+^ cells in the lungs from days 2 to 6 (14.9–28.9%, *P*<0.01). Not unexpectedly, cell death is accompanied by the release of cytosolic contents into the extracellular milieu. LDH activity was analyzed in the BALF recovered from *Ft* LVS-infected mice at different time points. Levels were substantially higher at day 6 than at day 2 or in uninfected mice (Figure 3c). HMGB1 also was abundantly detected in BALF (Figure 3d) and lung tissues (data not shown) at day 6 p.i.

### Characterization of cell death at the ultrastructural level

**Figure 4:**
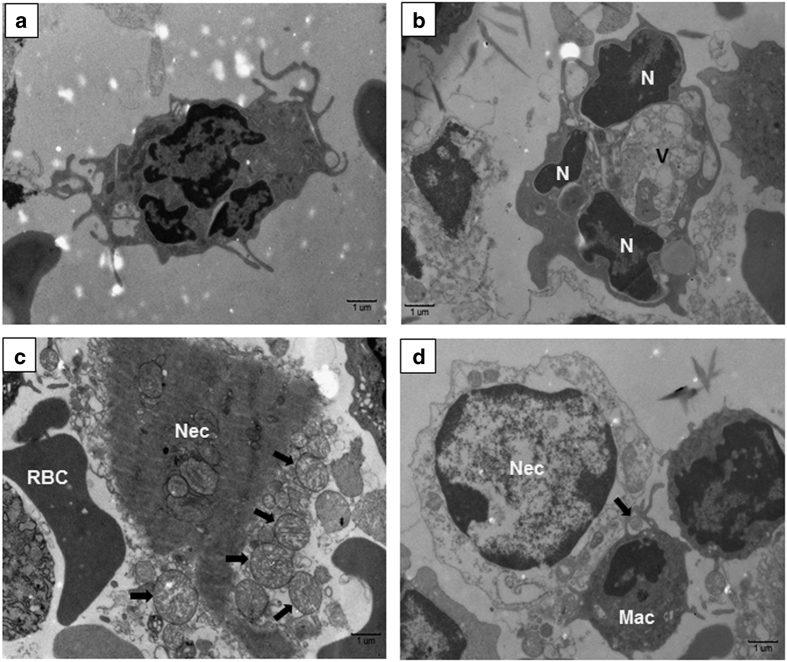
Electron microscopic analysis of leukocytes in normal and *Ft* LVS-infected lung. (**a**) Normal PMN isolated from sham-inoculated lung showing multilobed nucleus with normal distribution of chromatin structures. (**b**) Activated PMN exhibiting signs of apoptosis with condensed/marginated nuclear chromatin (N) and cytoplasmic vacuoles (v). (**c**) Necrotic cell (Nec) showing grossly swollen mitochondria being released into the extracellular space (arrows). Red blood cell, RBC. (**d**) Necrotic cell (Nec) being recognized by adjacent macrophage (Mac). Free mitochondrion (arrow) outside the cell appears to be phagocytized by a macrophage.

Next, we sought to characterize the cellular consequences of induction of apoptosis and necrosis at the ultrastructural level. Both apoptotic and necrotic leukocytes were observed, along with cellular debris including released, swollen mitochondria with abnormally round/dilated cristae (Figure 4). A quiescent cell, showing little or no activation, is typical of those very few PMNs recovered from uninfected lungs (Figure 4a). In contrast, PMNs and other leukocytes isolated from infected lungs were morphologically, highly activated and found to be in various stages of apoptosis/secondary necrosis (Figures 4b–d). Apoptotic cells demonstrated cytoplasmic and nuclear condensation, DNA damage, and formation of apoptotic bodies; however, the integrity of their plasma membrane remained intact (data not shown). By comparison, activated PMNs showed granular alterations and/or phagosomes and those undergoing secondary necrosis had condensed and electron-dense nuclei and ruptured cell membranes. In addition, some leukocytes also exhibited features of autophagy including vacuolization, degradation of cytoplasmic contents, and slight chromatin condensation. This observation is consistent with the ability of *Ft* to induce autophagy^29^ early during infection.^30^ Necrotic cell masses often showed grossly swollen, intact mitochondria and other cytosolic components being liberated into the extracellular milieu (Figure 4c). The release of intact, swollen mitochondria from cells undergoing RIP1-mediated necroptosis has been suggested by Maeda *et al*.^12^ Also evident was phagocytosis of mitochondria and other necrotic debris by adjacent macrophages (Figure 4d, inset).

### *Ft* triggers mitochondrial apoptosis

We interrogated various anti- and proapoptotic cell death markers in the cytoplasmic and mitochondrial fractions of cells. Infection with *Ft* SchuS4 resulted in significant changes in both antiapoptotic (Bax/Bcl-2, Mcl-1, Bcl-xl, and Bcl-xl/Bak) and proapoptotic markers (Bax, Bak, Bim, and caspase-3) (Supplementary Figures 1 and 2). Notably, antiapoptotic markers were found to be reduced in the mitochondrial fraction in *Ft*-infected lung homogenates while a significant increase in the proapoptotic markers was observed. Increases in Bax and Bak in the mitochondrial fraction of *Ft-*infected samples suggests translocation of Bax protein to the mitochondrial outer membrane, which is generally followed by the release of cytochrome *c* into the cytoplasm. Our results demonstrate that *Ft* SchuS4 triggers a Bax-dependent mitochondrial apoptotic pathway.

### Mitochondrial integrity is compromised in the lung of *Ft-*infected mice

**Figure 5:**
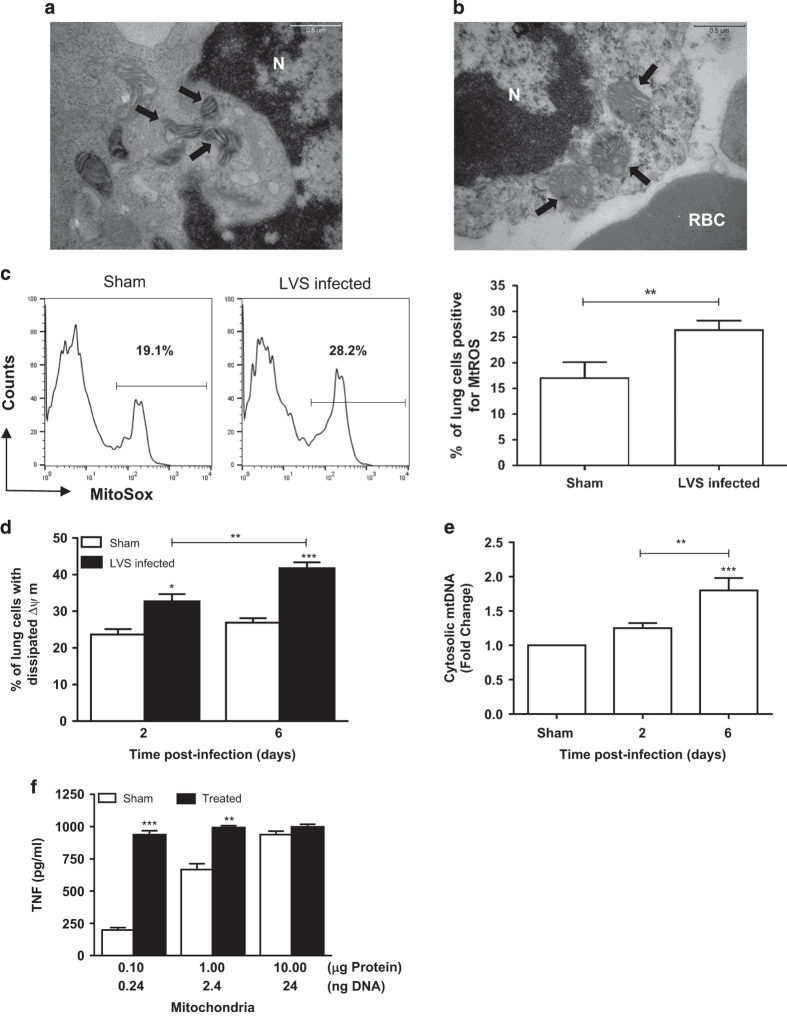
Damaged mitochondria in *Ft*-infected lungs represent a source of DAMPs that elicit T_H_1-type proinflammatory cytokines. (**a**) Electron micrograph of mitochondria (arrows) within a white blood cell isolated from sham-inoculated lung. Mitochondria have predominantly lamellar cristae. N, nucleus. (**b**) Electron micrograph of white blood cell isolated from infected lung tissue showing mitochondria (arrows) with abnormal, round/dilated cristae, some forming protrusions at the mitochondrial surface, and swollen size. RBC, red blood cell. (**c**) Superoxide levels in the mitochondria within lung cells from uninfected (Sham) and day 6 infected mice were determined by staining isolated cells with MitoSOX Red and were quantified by flow cytometry (left panel) as % cells positive for mitochondrial ROS (MtROS) (right panel). (**d**) The percentage of lung cells from uninfected (Sham) and *Ft*-infected mice whose mitochondria exhibit dissipated membrane potentials at various time points p.i. were quantified by flow cytometry using JC-1 staining. (**e**) Quantitative PCR analysis for the presence of mtDNA in the cytosol of lung cells from uninfected (Sham) and *Ft*-infected mice was performed as described in Materials and methods. (**f**) Mitochondria isolated from uninfected (Sham) and *Ft*-infected lungs at day 6 p.i. were evaluated for their proinflammatory capacity. Wild-type C57BL/6 BMDMs (2.5×10^5^ cells/well) were incubated with various amounts of mitochondria for 24 h. Supernatants were collected and assayed for the presence of TNF by ELISA (see also Supplementary Figure S2). Data are presented as the mean±S.E.M. from two to three independent experiments (*n*=6 mice per group or 12–18 mice total). *P<0.05, **P<0.01, and ***P<0.001. All results shown were subjected to one-way analysis of variance (ANOVA) with Bonferroni’s post-test.

Mitochondria are an important source of DAMPs; as such, we assessed whether mitochondria within and/or outside cells could trigger the T_H_1-oriented hypercytokinemia observed during late-phase tularemia. Electron microscopic analysis revealed a difference in the ultrastructure of mitochondria within cells recovered from uninfected (Figure 5a) *versus* *Ft* LVS-infected lungs at day 6 p.i. (Figure 5b). In contrast to ‘healthy’ mitochondria visualized in uninfected lung cells that have well-organized lamellar cristae and a normal size, those recovered from infected lungs had round/dilated cristae that were disorganized and the mitochondria were swollen in size (Figure 5b). In addition, swollen mitochondria from necrotic cells were free to migrate into the extracellular milieu as they were no longer bounded by an intact plasma membrane. A biochemical indicator of the general activation/metabolic state of mitochondria is the amount of superoxide produced. Superoxide levels in mitochondria recovered from mice infected for 6 days were significantly increased compared with sham-inoculated animals and indicative of increased oxidative stress (Figure 5c). Additionally, the number of MitoSOX-positive lung cells derived from *Ft*-infected mice was found to be significantly higher than that from sham-inoculated controls (3.2×10^6^±2.7×10^5^ *versus* 1.1×10^6^±1.7×10^5^, respectively; *P*<0.0001) (Figure 5c). Next, mitochondrial membrane potential (ΔΨm), which dissipates as mitochondria become progressively more damaged, was measured in lung cells derived from sham-inoculated and infected mice using a MitoProbe JC-1 Assay Kit (Molecular Probes, Eugene, OR, USA; Figure 5d). A significant increase in the percentage of lung cells with dissipated ΔΨm was seen in *Ft-*infected *versus* uninfected control lungs (32.60±2.10 *versus* 23.64±1.5, *P*<0.05) at day 2. An even greater dissipation of ΔΨm was observed at day 6 than at day 2 (*P*<0.01). We also determined whether diminished mitochondrial integrity was associated with leakage of mtDNA into the cytosol. Quantitative PCR was used to measure levels of mitochondrial *cytochrome c oxidase* DNA in cytosolic fractions generated from lung cells recovered from uninfected and *Ft-*infected mice. As shown in Figure 5e, the amount of mtDNA found in the cytosol of cells 2 days p.i. was not different than that in uninfected controls. However, by day 6 the amount of mtDNA found within the cytoplasm increased above background (*P*<0.001) and was significantly greater than that observed at day 2 (*P*<0.01).

### Isolated mitochondria trigger cytokine release by macrophages

To gain mechanistic insight into exactly what drives the hypercytokinemia observed during late-phase tularemia, we evaluated whether the damaged mitochondria isolated from infected lung tissue could elicit proinflammatory cytokines (that is, TNF). As seen in Figure 5f, addition of isolated mitochondria (representing as little as 0.24 ng of mtDNA or 0.1 *μ*g of total mitochondrial protein) to BMDMs triggered the release of ~1000 pg/ml of TNF; a response mammalian host-adapted *Ft* were unable to elicit even when added to cells at a ratio of 100 : 1 (10–20-fold above the *in vivo Ft*/leukocyte ratio).^20,21,31–33^ Isolated mitochondria from infected tissue also exhibited a dose-dependent capacity to stimulate significantly more IL-1*β*, IL-6, MCP-1, and KC from BMDMs than did mitochondria recovered from uninfected cells (Supplementary Figure 3). At very high mitochondria-per-cell ratios, the release of TNF in response to ‘healthy’ *versus* damaged mitochondria is indistinguishable (Figure 5f).

### *Ft* induces both caspase-dependent and -independent HCD in BMDMs

**Figure 6:**
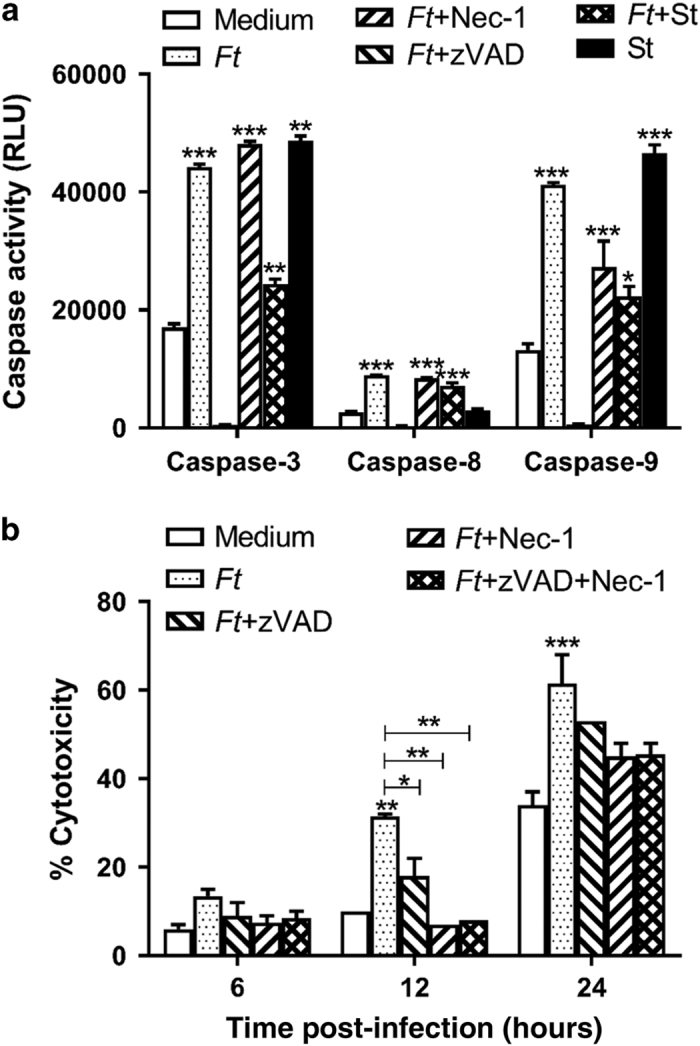
*Ft* infection induces cell death in a caspase-dependent and -independent manner. (**a**) C57BL/6 BMDMs were infected with *Ft* LVS at a multiplicity of infection (MOI) of 100 in the absence or presence of zVAD-fmk (20 *μ*M), necrostatin-1 (20 *μ*M), and staurosporine (1 *μ*M). Levels of active caspase-8, -9, and -3 were measured at 24 h p.i. (**b**) BMDMs were infected with *Ft* LVS at an MOI of 100 in the absence or presence of zVAD-fmk, necrostatin-1, or both. Lactate dehydrogenase (LDH) release was measured at the indicated time points. Data are presented as the mean±S.E.M. from three independent experiments. *P<0.05, **P<0.01, and ***P<0.001. All results shown were subjected to one-way analysis of variance (ANOVA) with Bonferroni’s post-test.

To investigate the role of caspases in cell death induced by *Ft*, we used a luminescence assay to measure the activity of caspase-3, -8, and -9. Caspase-3, an effector caspase, cleaves cellular targets that ultimately lead to apoptotic cell death, whereas caspase-8 and -9 are the initiator caspases and function as upstream activators of caspase-3. We observed that *Ft* infection of BMDMs activated both caspase-8 and -9, leading to the activation of caspase-3 (Figure 6a). All caspase activity was ablated by the addition of the pancaspase inhibitor, zVAD-fmk, while the presence of necrostatin-1, an inhibitor for necroptosis, did not have a significant effect. Interestingly, we observed that in the presence of *Ft*, caspase-3 and -9 activation induced by staurosporine was significantly reduced (*P*<0.001).

During *Ft* infection we observed that damaged mitochondria released into the extracellular milieu can be engulfed by macrophages. Since release of intact mitochondria is a feature of necroptotic cell death,^12^ we hypothesized that *Ft* may induce necroptosis. As seen in Figure 6b, the presence of zVAD-fmk and necrostatin-1 rescued a significant percentage of cells from death, especially at 12 h p.i. Collectively, these results suggest that *Ft*-infected BMDMs undergo both apoptotic and necroptotic death, which is caspase-dependent and -independent, respectively.

### *Ft*- infected macrophages undergo RIP1/3-dependent necroptosis

**Figure 7:**
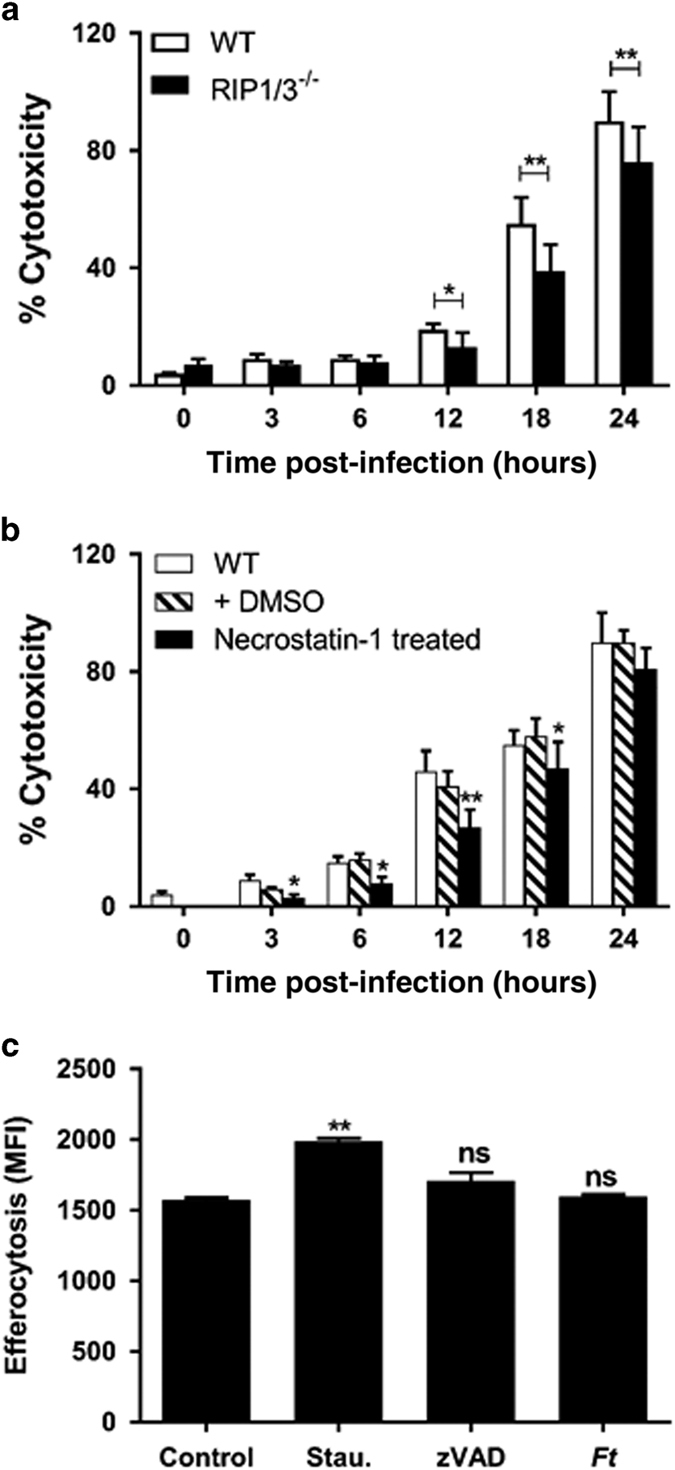
*Ft* induces necroptotic cell death in macrophages. (**a**) Wild-type (WT) and RIP1/3^−/−^ BMDMs were infected with *Ft* LVS at a multiplicity of infection (MOI) of 100 and lactate dehydrogenase (LDH) release was measured at the indicated time points. (**b**) WT BMDMs either were left untreated or were treated with dimethyl sulfoxide (DMSO) alone or necrostatin-1 (20 *μ*M) and LDH release was measured at the indicated time points. (**c**) WT BMDMs were treated for 24 h with medium alone or medium containing 5 *μ*M staurosporine (to induce apoptosis), or LPS+zVAD (to induce necroptosis) or with *Ft* alone. Dead cells were collected, counted, and resuspended in fresh medium and were applied to naïve BMDMs at a 2 : 1 ratio. After 2 h, cells were washed with cold phosphate-buffered saline (PBS) and the fluorescence intensity was quantified using a plate reader. Data are presented as the mean±S.E.M. from three independent experiments. *P<0.05, **P<0.01. All results shown were subjected to one-way analysis of variance (ANOVA) with Bonferroni’s post-test.

Given that necroptosis is mediated through a pathway that depends on the RIP1/3 complex, we used BMDMs from mice deficient for RIP1/3 to further test our hypothesis. As observed in Figure 7a, by 12 h p.i. *Ft* triggers significantly more cell death in the wild-type *versus* RIP1/3^−/−^ BMDMs. To seek confirmation of these results, we used necrostatin-1, a preferential inhibitor of the interaction between RIP1/3 and downstream effectors of necroptosis. We found significantly reduced cell death in the presence of necrostatin-1, which corroborates the observation made with RIP1/3^−/−^ cells (Figure 7b).

Since DAMPs were released through necroptosis during *Ft* infection and it has been demonstrated that macrophages more readily internalize apoptotic *versus* necroptotic cells,^34^ we questioned whether efferocytosis of these necroptotic cells is impaired. Impaired clearance of necroptotic cells from *Ft-*infected lungs would lead to further accumulation of DAMPs and thus perpetuate and exacerbate the cycle of tissue damage. To test this, we treated BMDMs with staurosporine (to induce apoptosis), LPS+zVAD (to induce necroptosis) or infected the cells with *Ft* (likely inducing necroptosis). Treated and infected cells were ‘fed’ to naïve BMDMs and their uptake was quantified. Significantly more apoptotic cells than necroptotic cells were engulfed by naïve BMDMs (Figure 7). The fact that uptake of *Ft*-infected cells was less than that of apoptotic cells and not statistically different from cells exposed to LPS+zVAD is consistent with our contention that *Ft* directly induces necroptosis.

## Discussion

Lung inflammation in response to infection with *Ft* LVS or SchuS4 is observed as early as 24–48 h p.i. and consists of both cellular (for example, PMNs and macrophages) and soluble (for example, IL-10, IL-17, MCP-1, KC, and TGF-*β*) elements. However, it is not until ⩾72 h that proinflammatory cytokines such as TNF, IL-1*β*, IL-6, IL-12p70, or IFN-*γ*, which are associated with sterilizing immunity and many other acute bacterial infections, are produced at significant levels. To better understand the unique kinetics of cytokine production and the ensuing sepsis-like syndrome, it is necessary to appreciate how *Ft* modulates HCD programs. Herein, we present evidence that the T_H_1 orientation of the hypercytokinemia or ‘cytokine storm’ associated with late-phase respiratory tularemia correlates with the onset of necroptosis and is a response to the release of DAMPs; in particular, damaged mitochondria whose accumulation is exacerbated by impaired efferocytosis. Using a murine respiratory model of *Ft* infection our study suggests that not only were liberated mitochondria observed in the extracellular milieu of the inflamed lung but they were phagocytized by macrophages along with other necrotic debris. Moreover, mitochondria released during *Ft* infection were highly proinflammatory in nature and have a unique ability to elicit TNF, IL-1*β*, IL-6, MCP-1, and KC from macrophages, a capacity *Ft* itself lacks.^20,21^

It has been suggested that *in vivo*, necroptosis elicits an intense inflammatory reaction;^8,35^ however, there is limited understanding of the molecular mechanisms involved and the role and nature of the proinflammatory DAMPs released during necroptosis, especially in the context of infectious diseases. Active release of intact organelles from cells during necroptosis represents a ‘danger signal’ that perpetuates inflammation.^12^ We report here, to our knowledge for the first time, that during *Ft* infection mitochondria are released from cells undergoing RIP1/3-dependent necroptosis. As reviewed by Krysko *et al.*,^36^ the proinflammatory capacity of damaged mitochondria within cells undergoing apoptosis/secondary necrosis increases as their structural integrity degrades, superoxide generation rises, and mtDNA and proteins are released into the cytosol and, ultimately, into the extracellular environment.^37^ A combination of electron microscopy, flow cytometry, and biochemical analyses of mitochondria demonstrated that all of these pathologic changes occur in response to infection with *Ft.* This suggests that *Ft*-induced necroptosis and subsequent release of mitochondria engenders the septic-like environment associated with late-phase tularemia.

In addition to release of mtDAMPs, insufficient autophagy of damaged mitochondria as well as impaired efferocytosis of dying and dead cells can lead to the release of a diverse array of non-mitochondrial DAMPs (for example, HMGB1, S100 proteins, IL-33 etc.).^8^ Our finding that the cytokine ‘storm’ coincides with *Ft* LVS-induced increases in HMGB1 after day 3 is consistent with those reported by Sharma *et al*.,^18^ for *Ft* SchuS4. Limited if any earlier release of such DAMPs during acute-phase tularemia (<48 h) may be explained by the ability of *Ft* to delay induction of apoptosis in human PMNs^38^ and the ability of TolC protein secreted by *Ft* LVS to delay activation of the intrinsic apoptotic pathway in macrophages during the first 36 h of infection.^17^ McCracken *et al*.^39^ reported that *Ft* delays the induction of apoptosis in human neutrophils by inhibiting Bax translocation and Bid processing, which sustains mitochondrial integrity, and by activating X-linked inhibitor of apoptosis protein and proliferating cell nuclear antigen, which inhibit caspase-9 and -3 by direct binding.^39^ Consistent with these reports, it was observed that *Ft* infection of BMDMs inhibited caspase activation triggered by the potent proapoptotic agent staurosporine. Despite such delays these cells eventually undergo apoptosis later in the disease process^1,40^ and then are ‘susceptible’ to signals that trigger necroptosis.

Typically, necroptosis ensues following TNF-mediated TNFR1 ligation, which follows activation of RIP1, RIP3, or MLKL (that is, mixed lineage kinase domain-like kinase).^8^ Although necroptosis has been studied extensively from the perspective of sterile inflammation,^12^ only recently has its significance in the context of acute bacterial infections been explored. Recently, *Salmonella* Typhimurium was reported to drive RIP1/3-dependent necroptosis, which facilitates evasion of the immune response by the pathogen.^35^ Our current findings suggest that *Ft*-infected macrophages undergo RIP1/3-dependent necroptosis. What is yet to be fully explained is how this mode of programmed HCD is initiated in the absence of TNF production or TNFR1 stimulation during the early phase (<72 h) of tularemia. There is, however, precedent insofar as necroptosis occurs independent of TNF in the context of infection with *S.* Typhimurium as well.^35^ Finally, the importance of necroptosis as a fundamental aspect of tularemia pathogenesis is supported by the findings that necrostatin-1 treatment of *Ft-*infected mice results in significantly decreased lung bacterial burden and pathology scores, and increased survival following lethal challenge.^41^

**Figure 8:**
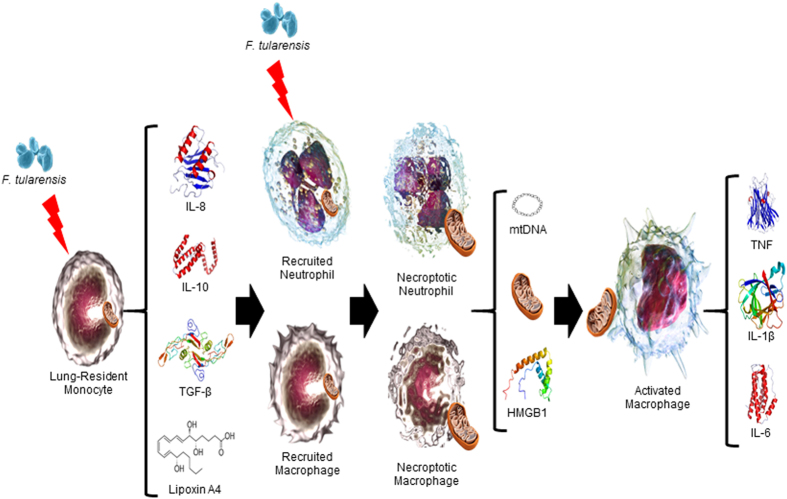
Schematic representation of *F. tularensis*-induced generation of mitochondrial DAMPs and sepsis-like syndrome. Initial infection of lung-resident cells by *F. tularensis* results in a principally anti-inflammatory response, typified by production of interleukin-10 (IL-10), tumor growth factor-*β* (TGF*β*), and lipoxin A4 (LXA_4_). However, simultaneous release of potent chemokines (for example, keratinocyte-derived chemoattractant (KC)/IL-8 and monocyte chemotactic protein-1 (MCP-1)), whose production is augmented by LXA_4_, drives the recruitment of PMNs and macrophages that support exponential replication of bacteria following their infection. *F. tularensis*-induced delay in apoptosis of these infected cells gives way to caspase-3-mediated, RIP1/3-dependent necroptosis and subsequent release of mitochondria and other DAMPs, of both mitochondrial and non-mitochondrial origin. A target of such DAMPs are activated macrophages that secrete the proinflammatory cytokines that can result in end-organ failure and death.

Taken together, our studies point to important crosstalk between necroptosis, release of mitochondrial and non-mtDAMPs, and the severe inflammation observed during the late-phase of respiratory tularemia (Figure 8). The consequent T_H_1-oriented inflammatory response to these DAMPs then mediates more effective bacterial clearance; however, from the host’s perspective it is ‘too little too late’. These findings are of particular interest because successful identification of the bacterial component(s) and mechanisms responsible for induction of TNF-independent necroptosis will extend our understanding of this unique mode of cell death and perhaps identify druggable targets for the development of therapeutics.

## Materials and methods

### Ethics statement

This study was conducted in strict accordance with the recommendations in the Guide for the Care and Use of Laboratory Animals of the National Institutes of Health. The protocols were approved by the Institutional Animal Care and Use Committees of Albany Medical College and Southern Research.

### Bacteria

*Ft* LVS was kindly provided by Dr. Karen Elkins (US FDA, Bethesda, MD, USA). *Ft* SchuS4 was obtained from the USAMRIID (Frederick, MD, USA). Bacteria were cultured in Muller–Hinton II broth or brain heart infusion broth (BHIB) as described.^20,21^

### Animal infection

C57BL/6 mice, purchased from National Cancer Institute (Frederick, MD, USA), were housed in the Animal Resources Facility at AMC or SR. Food and water were provided *ad libitum* and mice were infected as described previously.^20,21^ All infection experiments used groups of 6–10 mice that were monitored for survival or killed at designated times p.i. To date, no gender bias in the innate immune mechanisms under investigation have evinced themselves. Following dilution in sterile BHIB, 1×10^3^ CFU of *Ft* LVS or 2×10^1^ CFU of *Ft* SchuS4 in a volume of 20 *μ*l was instilled dropwise (10 *μ*l per nare). Actual dosages received by the mice were confirmed by colony plating. Sham-inoculated controls received an equal volume of sterile BHIB. killed mice were necropsied at various times p.i. and lungs were perfused with PBS and excised aseptically. The smaller lobe of the lung was used for preparation of lung homogenate for immunomodulator analysis and determination of bacterial burden as described previously. BALF samples were collected and stored at −20 °C. Organs were excised for histological evaluation as described previously^28^ and to isolate cells and mitochondria for flow cytometric and biochemical analysis. For survival experiments, mice were examined two times daily for morbidity and mortality for a period of 21 days and the mean time to death and median survival was calculated for each group.

### Immunohistochemistry

Lung samples were processed as described previously^28^ and stained using hematoxylin–eosin or the TUNEL method. TUNEL staining was performed using the *In situ* Cell Death Detection Kit, TMR Red (Roche Applied Science, Indianapolis, IN, USA).

### Characterization of apoptosis and necrosis of lung cells

Multiparameter flow cytometry was used to analyze lung cells that were stained for myeloid lineage antigens and gated on specific phenotypic surface markers. Based on the expression of phenotypic markers, myeloid cells in the lungs were identified as PMNs (CD11BHIB^gh^GR1^high^F4/80^low^CD11c^low^) and macrophages (CD11BHIB^gh^GR1^low^F4/80^high^CD11c^low^). Unless otherwise stated, all lungs were perfused with PBS before isolation of cells. These cells then were stained using the DeadEnd Fluorometric TUNEL System (Promega Corp., Madison, WI, USA) and analyzed by flow cytometry for apoptosis. Necrosis was evaluated by staining cells with 7-AAD (0.25 *μ*g/10^6^ cells) (BD Pharmingen, San Diego, CA, USA) for 10 min at room temperature and were analyzed by flow cytometry.

### LDH release assay and HMGB1 ELISA

Cell death was quantified using a CytoTox96 LDH Release Kit (Promega). HMGB1 levels were measured in BALF samples and lung homogenates were isolated from *Ft-*infected mice using a specific ELISA Kit (IBL International GmbH, Hamburg, Germany) according to the manufacturer’s protocol.

### Caspase activity assay

Caspase-3, -8, and -9 assays were performed by using Caspase-Glo 3/7, Caspase-Glo 8, and Caspase- Glo 9 Assay Kits respectively (Promega) following the manufacturer’s protocol.

### Apoptosis luminex assay

Samples derived from *Ft*-infected mice were analyzed for the presence of proapoptotic and antiapoptotic markers following the manufacturer’s instructions (Bio-Rad Laboratories, Hercules, CA, USA).

### Isolation of BMDMs

BM progenitor cells were isolated from femurs to enrich for BMDMs as described previously.^20,21^

### Transmission electron microscopy

Lung cells were isolated as described above and fixed in equal volumes of cold 2.5% glutaraldehyde and 4% formaldehyde. The fixed cells were washed two times with cold PBS and treated with 1% osmium tetroxide for 1 h at 4 °C. Cells were dehydrated in an ethyl alcohol series and then cleared and embedded in Spurr's resin. Sections were cut using a Reichert Ultracut E ultramicrotome (Buffalo Grove, IL, USA), placed on nickel hexagonal grids with carbon-coated formvar support films, and stained with uranyl acetate for 30 min, washed, and Reynold's lead for 10 min. Images were obtained using a Zeiss 910 transmission electron microscope (Oberkochen, Germany) at 80 kV.

### Immunomodulator quantification

Cytokine/chemokine levels in tissue culture supernatants were measured by Mouse Inflammation Cytometric Bead Array Kit or individual Flex Sets (BD Pharmingen). Flow cytometric analysis was performed using a FACSArray flow cytometer (BD Immunocytometry Systems (BDIS), San Jose, CA, USA). Data were acquired and analyzed using BD FACSArray Software and FCAP Array software, version 1.0 (BDIS), respectively.

### Isolation of mitochondria and characterization of mitochondrial ‘health’ and cell-stimulatory activity

Perfused lungs were isolated from uninfected and infected mice at various times p.i., minced, and enzymatically digested to recover total lung cells as described above. The ∆ψm of isolated lung cells was measured using a MitoProbe JC-1 Assay Kit (Molecular Probes). Mitochondrial superoxide levels were determined within the mitochondria of isolated lung cells using a MitoSOX Red Mitochondrial Superoxide Indicator Kit (Molecular Probes). Mitochondria were isolated from uninfected and infected lung tissue using a Mitochondrial Isolation Kit for Tissues or the kit specific for cells (Thermo Scientific, Rockford, IL, USA). To evaluate their cell-stimulatory capacity, increasing amounts of mitochondria (based on total protein and mtDNA concentration) were incubated with wild-type BMDMs for 24 h. Cytokine and chemokine levels in tissue culture supernatants were measured by ELISA and/or Cytometric Bead Array as described above.

### Quantitative real-time PCR

The amount of mtDNA released into the cytosol of lung cells recovered from uninfected and infected mice was quantified as described elsewhere.^42^

### Efferocytosis assay

Efferocytosis assays were performed using BMDMs as described previously.^34^ Following treatment of BMDMs with or without 5 *μ*M staurosporine (to induce apoptosis) or LPS (200 ng/ml)+100 *μ*M zVAD (to induce necroptosis) for 24 h, Vybrant dye (5 *μ*M; Invitrogen, Waltham, MA, USA) was added for 30 min at 37 °C to label the cells. Labeled cells then were added to monolayers of naïve unlabeled BMDMs at a ratio of 2:1. After 2 h incubation, efferocytosis of the labeled cells by naïve cells was calculated as a proportion of uptake of labeled cells by quantifying fluorescence intensity using a plate reader.

### Statistical analysis

Where applicable, all results were expressed as mean±S.E.M. from two or more independent experiments. Depending upon the distribution of the data set, comparisons between groups were made using a parametric ANOVA test with Bonferroni’s post-test or a nonparametric Kruskal–Wallis test with Dunn’s post-test. Differences between control and experimental groups were considered significant at *α*=0.05 level.

## Additional information

**Publisher’s note:** Springer Nature remains neutral with regard to jurisdictional claims in published maps and institutional affiliations.

## Figures and Tables

**Table 1:** Total number of specific cell types undergoing cell death at day 3 in *Ft* LVS-infected mice

*Cells*	*TUNEL*^*+*^ *cells*		*7-AAD*^*+*^ *cells*	
	*Sham*	*Infected*	*Sham*	*Infected*
PMN	1.013±408	149.483±58.681^a^	373±77	37.345±4.885^a^
Mφ	20.677±2.260	243.311±19.116^a^	8.978±2.027	108.510±26.668^b^

Abbreviations: *Ft*, *Francisella tularensis* ssp. *tularensis*; 7-AAD, 7-aminoactinomycin D; LVS, live vaccine strain; PMN, polymorphonuclear leukocytes; TUNEL, terminal deoxynucleotidyl transferase dUTP nick-end labeling.

^a^*P*<0.05. ^b^*P*<0.01.

**Table 2:** Total number of specific cell types undergoing cell death at day 3 in *Ft* SchuS4-infected mice.

*Cells*	*TUNEL*^*+*^ *cells*		*7-AAD*^*+*^ *cells*	
	*Sham*	*Infected*	*Sham*	*Infected*
PMN	389±121	80.791±37.120^a^	6.565±5.464	132.781±56.543^b^
Mφ	30.868±5.820	32.413±6.792	19.597±2.765	126.474±36.391^a^

Abbreviations: *Ft*, *Francisella tularensis* ssp. *tularensis*; 7-AAD, 7-aminoactinomycin D; PMN, polymorphonuclear leukocytes; TUNEL, terminal deoxynucleotidyl transferase dUTP nick-end labeling.

^a^*P*<0.001. ^b^*P*<0.01.
