# Supplementary material for: Necroptotic debris including damaged mitochondria elicits sepsis-like syndrome during late-phase tularemia
Source: Cell Death Discov. 2017 Sep 25;3:17056–. doi: 10.1038/cddiscovery.2017.56 (PMC5611684; doi:10.1038/cddiscovery.2017.56)
Supplement: Supplementary Figure Legends [file cddiscovery201756-s1.pdf]

Supplementary Figure 1. Detection of anti-apoptotic biomarkers during *Ft*-infection. C57BL/6 mice were infected with 20 CFU of SchuS4 and the lungs were processed at day 3 p.i. to isolate the cytosolic and mitochondrial fraction. The levels of various anti-apoptotic biomarkers such as Bcl-xl/Bak (a); Bcl-xl (b); Bax/Bcl-2 (c); and Mcl-1 (d) were analyzed in the both the cytosolic and mitochondrial fraction. Values in the parentheses represent the fold change in the specific pro- and anti-apoptotic markers present in the cytosolic fraction vs. the mitochondrial fraction. Data are presented as the mean ± SEM from two independent experiments (n = 6 mice per group or 12 mice total). *$P < 0.05$, **$P < 0.01$ and ***$P < 0.001$. All results shown were subjected to One-way ANOVA with Bonferroni's Post-test.

Supplementary Figure 2. Detection of pro-apoptotic biomarkers during *Ft*-infection. C57BL/6 mice were infected with 20 CFU of SchuS4 and the lungs were processed at day 3 p.i. to isolate the cytosolic and mitochondrial fraction. The levels of various pro-apoptotic biomarkers such as Bax (a); Bak (b); Bim (c); and Caspase-3 (d) were analyzed in the both the cytosolic and mitochondrial fraction. Values in the parentheses represent the fold change in the specific pro- and anti-apoptotic markers present in the mitochondrial fraction vs. the cytosolic fraction. Data are presented as the mean ± SEM from two independent experiments (n = 6 mice per group or 12 mice total). *$P < 0.05$, **$P < 0.01$ and ***$P < 0.001$. All results shown were subjected to One-way ANOVA with Bonferroni's Post-test.

Supplementary Figure 3. Damaged mitochondria elicit production of a variety of pro-inflammatory mediators from BMDMs. Mitochondria isolated from lungs at day 6 p.i. with *Ft* LVS were evaluated for their pro-inflammatory capacity. Wild-type C57BL/6 BMDMs (2.5×10^5 cells/well) were incubated with various amounts of mitochondria for 24 h. Supernatants were collected and assayed for the presence of IL-1β (a), IL-6 (b), MCP-1 (c), and KC (d) by CBA. Data are presented as the mean ± SEM from two independent experiments. *$P < 0.05$, **$P < 0.01$, and ***$P < 0.001$. All results shown were subjected to One-way ANOVA with Bonferroni's Post-test.
