# Supplementary figures and images for: Necroptotic debris including damaged mitochondria elicits sepsis-like syndrome during late-phase tularemia
Source: Cell Death Discov. 2017 Sep 25;3:17056–. doi: 10.1038/cddiscovery.2017.56 (PMC5611684; doi:10.1038/cddiscovery.2017.56)

Supplementary Figure 1

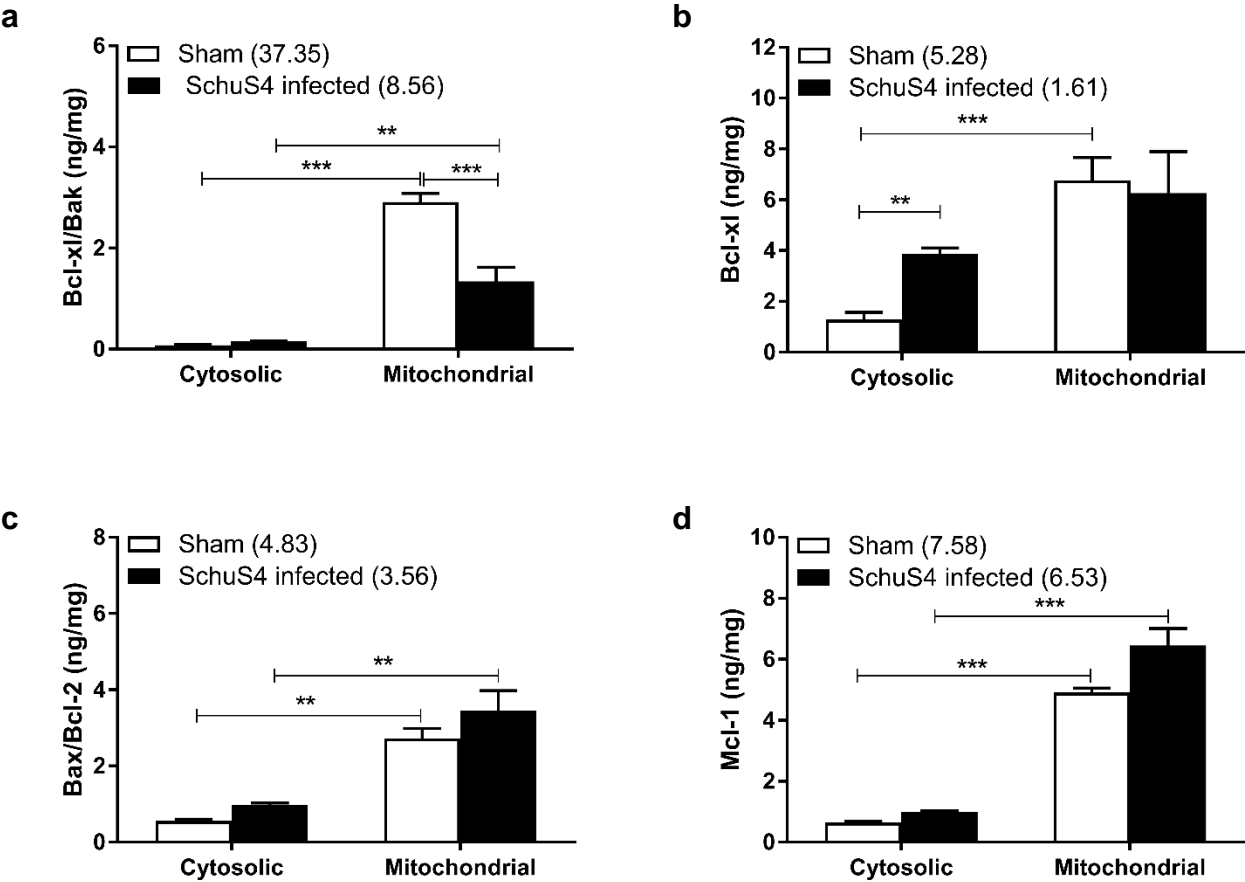

Supplementary Figure 2

a

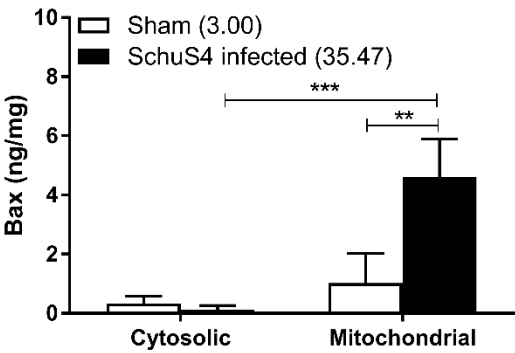

b

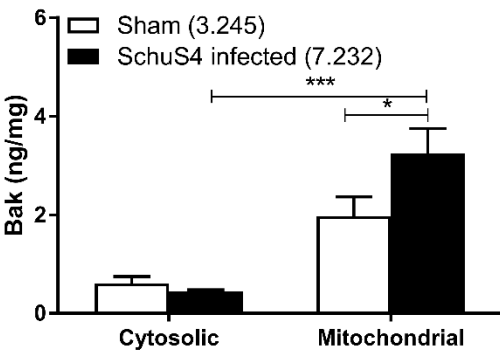

c

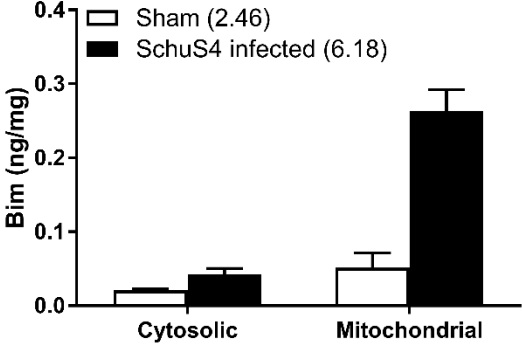

d

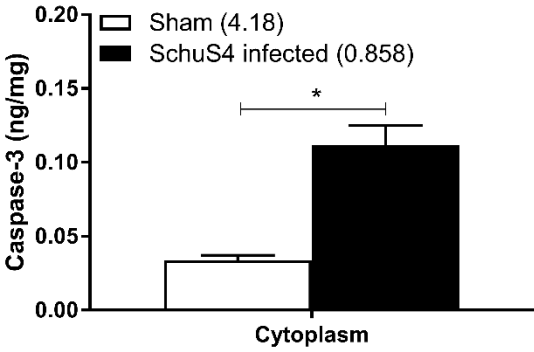

## Supplementary Figure 3

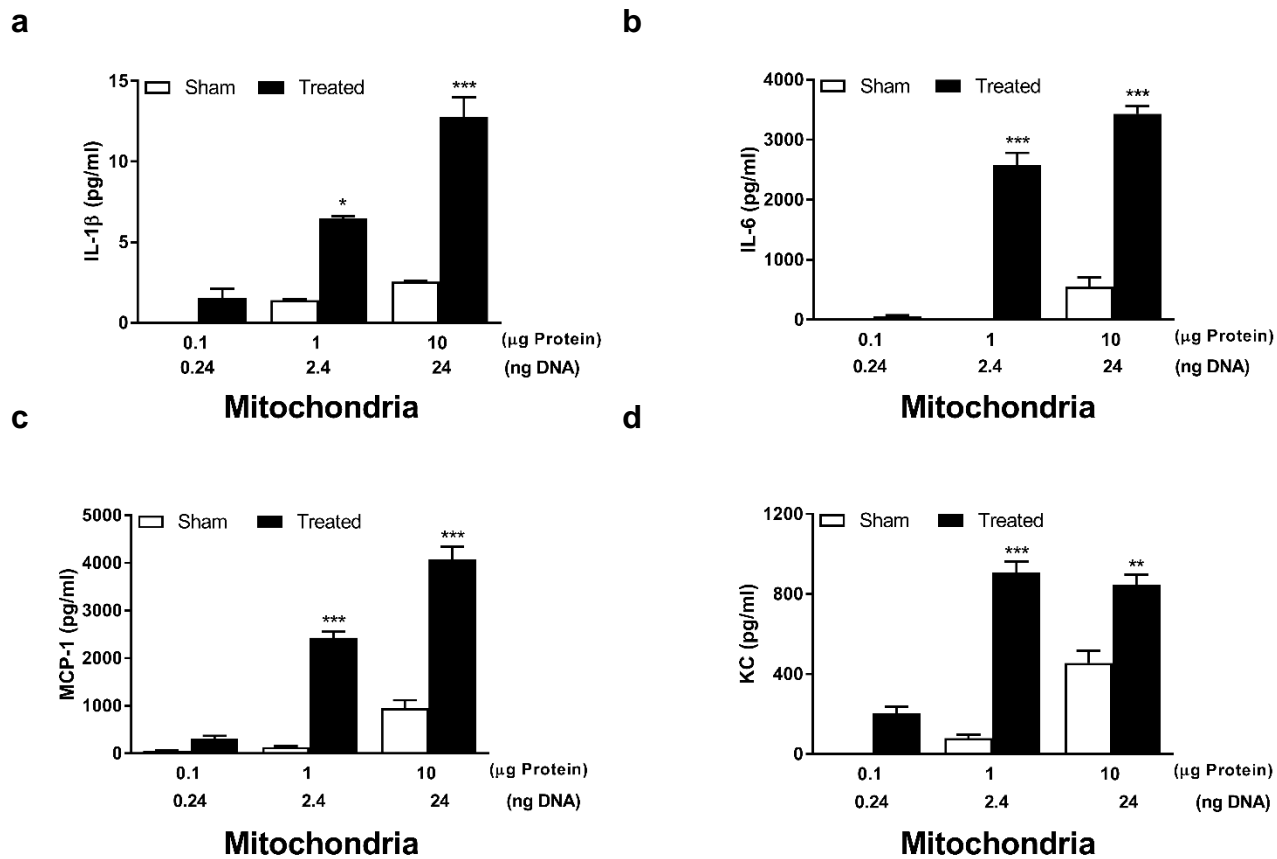

Supplement: Supplementary Figures [file cddiscovery201756-s2.pdf]
